# Analysis of Regulatory Network Involved in Mechanical Induction of Embryonic Stem Cell Differentiation

**DOI:** 10.1371/journal.pone.0035700

**Published:** 2012-04-27

**Authors:** Xinan Zhang, Maria Jaramillo, Satish Singh, Prashant Kumta, Ipsita Banerjee

**Affiliations:** 1 School of Mathematics and Statistics, Central China Normal University, Wuhan, China; 2 Department of Chemical Engineering, University of Pittsburgh, Pittsburgh, Pennsylvania, United States of America; 3 Department of Bioengineering, University of Pittsburgh, Pittsburgh, Pennsylvania, United States of America; 4 Department of Mechanical Engineering and Materials Science, University of Pittsburgh, Pittsburgh, Pennsylvania, United States of America; 5 Center for Complex Engineered Multifunctional Materials, University of Pittsburgh, Pittsburgh, Pennsylvania, United States of America; Baylor College of Medicine, United States of America

## Abstract

Embryonic stem cells are conventionally differentiated by modulating specific growth factors in the cell culture media. Recently the effect of cellular mechanical microenvironment in inducing phenotype specific differentiation has attracted considerable attention. We have shown the possibility of inducing endoderm differentiation by culturing the stem cells on fibrin substrates of specific stiffness [1]. Here, we analyze the regulatory network involved in such mechanically induced endoderm differentiation under two different experimental configurations of 2-dimensional and 3-dimensional culture, respectively. Mouse embryonic stem cells are differentiated on an array of substrates of varying mechanical properties and analyzed for relevant endoderm markers. The experimental data set is further analyzed for identification of co-regulated transcription factors across different substrate conditions using the technique of bi-clustering. Overlapped bi-clusters are identified following an optimization formulation, which is solved using an evolutionary algorithm. While typically such analysis is performed at the mean value of expression data across experimental repeats, the variability of stem cell systems reduces the confidence on such analysis of mean data. Bootstrapping technique is thus integrated with the bi-clustering algorithm to determine sets of robust bi-clusters, which is found to differ significantly from corresponding bi-clusters at the mean data value. Analysis of robust bi-clusters reveals an overall similar network interaction as has been reported for chemically induced endoderm or endodermal organs but with differences in patterning between 2-dimensional and 3-dimensional culture. Such analysis sheds light on the pathway of stem cell differentiation indicating the prospect of the two culture configurations for further maturation.

## Introduction

Lineage specific differentiation of embryonic stem cells (ESC) can have a tremendous impact on the therapeutic treatment of various degenerative diseases. Research over the last decade has established the possibility of differentiating ESCs *in-vitro* to many organ specific cell types [2]. Most commonly, *in-vitro* inductions of differentiation are achieved through modulations of the cellular chemical microenvironment by adding specific growth factors, inducers or repressors. More recently, the effect of mechanical cues such as substrate stiffness on differentiation is being investigated. Mesenchymal stem cells, when cultured on substrates of varying stiffness were reported to exhibit significant difference in their lineage commitment, which could be correlated to the physiological stiffness of the differentiated phenotype [3]. In our experience with embryonic stem cells, we have also observed such stiffness-specific differentiation of embryonic stem cells, where we reported the effect of variation of fibrin gel properties on early germ layer commitment of the ESCs [1]. Mouse embryonic stem cells (mESC) were cultured on fibrin gels fabricated under various fibrinogen and thrombin concentrations, which resulted in variation of gel stiffness in the range of 4 Pa – 247 Pa. These experiments were conducted in two different cell culture configurations: cells seeded on top of pre-formed 2D fibrin gels as well as cells embedded inside the 3D fibrin gels. Under both conditions it was observed that gels with stiffness values in the lower range (4 Pa – 14 Pa) preferentially favors stem cell commitment towards endoderm germ layer, whereas the mesoderm and ectoderm markers where relatively insensitive to gel stiffness in the examined range. It is worth mentioning that no other endoderm specific induction was used in the culture media in order to ensure that the observed effect is solely from cell-substrate interaction. While endodermal differentiation was confirmed by specific gene and protein markers, it will be useful to analyze the regulatory network involved in the process of mechanical induction of germ layer. Until now all of the existing protocols rely on chemical induction of endoderm primarily through Activin (Tgfb) pathway; adaptation of an alternate mode of differentiation will benefit from an evaluation of potential regulatory mechanisms activated in the process.

In this paper we are investigating such network interaction activated during endoderm specification of ESC by mechanical induction from the substrate. Mouse ESCs were cultured on the fibrin gels fabricated under different conditions for 4 days, at the end of which they are analyzed in detail for various endoderm specific markers. Hence the data which we gather from the experiment consists of a matrix of relative expression of endoderm specific genes across various substrate conditions. Our objective here is to capture the regulatory architecture of the system from this gene-condition data set. One avenue in achieving this is through identification of subsets of genes which are exhibiting similar activation trends under multiple stimulatory conditions. The underlying assumption here is that if specific genes are highly co-expressed over a range of different conditions, their activation is probably related through a network, and hence can be considered to be participating in the same regulatory pathway. This class of problem can be handled by a technique called bi-clustering, which enables identification of subsets of genes exhibiting similar trends in expression levels over specific experimental conditions.

### Bi-clustering

Bi-clustering, which can be viewed as two-dimensional clustering, identifies subsets of genes which are similarly expressed across specific subsets of experimental conditions. Compared to clustering which applies to a single direction, biclustering can group both genes and conditions simultaneously. The motivation behind this technique comes from the understanding that specific regulatory networks, consisting of specific transcription factors, can be activated under certain experimental conditions only. Hence of all the genes and conditions examined only a subset of genes will be co-expressed under subset of experimental conditions. On the other hand the same gene can be participating in more than one network, which can be activated under different conditions. Hence it is entirely feasible to identify multiple biclusters from a single gene-condition data set, with some overlapping among different bicusters. While parallel techniques like Gene Set Enrichment Analysis (GSEA) [4] has been widely used to determine significantly differentially expressed genes, this method is mostly applied when we have some information about gene functions and gene relationships. Hence GSEA is sometimes used in conjunction with bi-clustering, where the bi-clustered sets are further analyzed using GSEA typically between two different states [5], [6].

The technique of bi-clustering was first introduced by Hartigan [7], under the name of “direct clustering”, with the goal of finding bi-clusters with minimum variance. Cheng and Church [8] further formalized the concept in the context of gene expression data by using residue of an element and the mean squared residue of a sub-matrix. In biological terms the residue is a measure of the similarity of gene expression trends between different conditions. However this measure will also identify genes exhibiting minimal dynamics across conditions. Such trivial bi-clusters were rejected by means of maximizing row variance, which ensures that the genes are exhibiting sufficient dynamics in their expression. Alternate approaches to bi-clustering have also been proposed by Getz et al. [9] applying hierarchical clustering separately to each dimension, thereby creating a coupled two-way clustering. Another approach is pattern-based clustering, that captures the similarity of the patterns exhibited by a bi-cluster [10]. While the bi-clustering formulation proposed by Cheng and Church [8] is most commonly used across fields, there is great diversity in the solution procedure adopted by different groups [11], [12], [13], [14].

Bi-clustering has been identified as NP-hard [15] and often it is solved via heuristics. Heuristics however, have its limitation in often identifying sub-optimal bi-clusters and being unable to identify arbitrarily overlapped bi-clusters [16]. In identification of transcription factor networks it will be important to identify overlapped bi-clusters, which allows identification of transcription factors participating in multiple pathways. Recently, an alternate approach has been proposed in formulating bi-clustering as an optimization problem [16], [17]. The overall objective in this formulation remains similar to the original bi-clustering concept [8]: identifying sub-matrices of maximum volume, having low residue while retaining high variance. In this paper we have adopted the solution procedure proposed by Divina [16] in identifying subsets of genes co-regulated over specific substrate conditions.

### Handling Data Variability

The system of embryonic stem cell is known for its heterogeneity and stochasticity. Differences among biological repeats can occur in these cultures because of the use of different passages of ES cells or by spontaneous differentiation, leading to substantial variation in between cells while still retaining similar trend towards specific differentiated phenotype [18].

Hence robust mathematical analysis of the system becomes challenging and often unreliable because of the uncertainty in the experimental data. It will thus be important to evaluate the variability of bi-clustering results based on the observed dataset. One way to estimate the variability is to evaluate a large number of experimental replicates and perform the bi-clustering algorithm over the entire data set. This is however an impractical option and *bootstrapping* provides a mathematical analog of a similar concept without the need for large experimental data sets.

The essence of bootstrapping lies in utilizing limited sampled data in deriving statistically significant parameters [19], [20]. A larger pseudo dataset is generated using the sampled dataset by re-sampling with replacement technique. The technique of bootstrapping was originally presented systematically by Efron [21]. A significant body of bootstrapping literature deals with estimating parameter variances and confidence intervals. Bootstrap techniques have thus far evolved into myriads of biological applications, in the areas of ecology, genetics and environmental science and engineering to name a few. In the current project we apply bootstrapping technique in order to determine a robust group of co-regulated genes identified through bi-clustering of the experimental data. To the best of our knowledge this is the first attempt in applying the bootstrap technique in the area of bi-clustering.

## Results

### Effect of Substrate Stiffness on Endodermal Gene Expression

**Figure 1:**
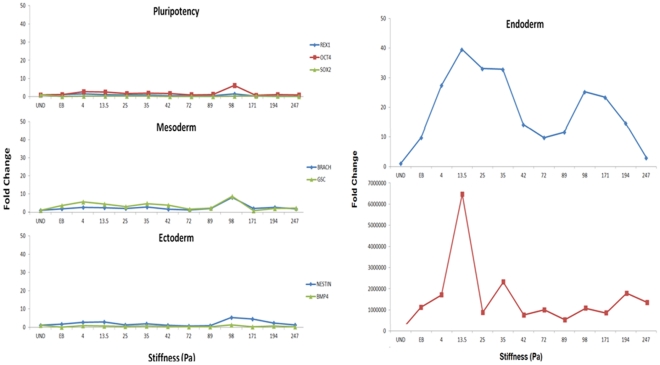
Effect of stiffness of fibrin substrate on early differentiation patterning of mouse embryonic stem cell. Embryonic stem cells were differentiated for 4 days on fibrin substrates of varying properties. Analysis of the differentiated cells for pluripotency and germ layer markers at the end of differentiation reveals that pluripotency, mesoderm and ectoderm markers are relatively insensitive to changes in substrate stiffness. The endoderm markers, specifically Sox17 and AFP responded strongly to the changes in stiffness in the chosen range. A lower value of stiffness resulted in stronger up-regulation of endoderm marker. The above analysis is for 3D culture configuration. In order to evaluate the effect of the substrate relative to chemical induction, a control experiment of spontaneous differentiation by embryoid body formation was performed, depicted by EB in the above plots. Spontaneous differentiation by EB formation typically resulted in lower upregulation compared to induction by fibrin substrate.

The system we are presenting in this paper is the effect of mechanical property of the substrate on germ layer induction of embryonic stem cells. In particular, we are concentrating on the stiffness modulus of the substrate. Fibrin was used as the substrate, whose properties were modified by changing either the fibrinogen concentration or the fibrinogen/thrombin cross-linking ratio. A broad range of storage moduli was obtained ranging from 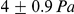 to 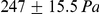 by varying the fibrinogen concentrations from 1, 2, 4 and 8 mg/ml, while maintaining the fibrinogen to thrombin ratio at 0.25x, 1x and 2x for each of the four fibrinogen concentrations. Details of the concentrations used and the substrate stiffness values of each substrate component are presented in Table 1. The experiments were performed under two different culture conditions: 2-dimensional (2-D), where the embryonic stem (ES) cells were cultured on top of pre-formed gels and 3-dimensional (3-D) where the ES cells are embedded inside the fibrin gel. The cells were differentiated on these substrates for 4 days, at the end of which the samples were collected and analyzed for relevant gene expression levels. It was interestingly observed that while mesoderm and ectoderm markers were relatively insensitive to changes in substrate stiffness, the endoderm markers elicit a strong response, having a strong expression under lower substrate stiffness conditions in the range of 4 Pa – 14 Pa [1] (Figure 1). Both the 2-D and 3-D culture showed similar effect of endoderm differentiation, although the effect in 3-D culture was much stronger than 2-D. In 2-D culture the differentiating cells were uniformly exposed to the media, which evidently was not the case under 3-D because of likely differences in diffusivity linked with variations in substrate properties. In order to test for the effect of media alone on differentiation we performed another control experiment where the ESCs were differentiated into embryoid bodies (EB) through hanging drop method. These EBs when analyzed for the germ layer markers showed only a subtle upregulation relative to the substrate mediated induction; indicating the media to be less dominant in differentiation induction.

**Table 1:** Fibrinogen and thrombin concentration used to synthesize the gel and corresponding stiffnessvalues.

		Thrombin Crosslinking		
	(a)	**0.25X**	**1X**	**2X**
	**1mg/ml**	0.1	0.4	0.8
	**2mg/ml**	0.2	0.8	1.6
	**4mg/ml**	0.4	1.6	3.2
	**8mg/ml**	0.8	3.2	6.4
**Fibrinogen** **Concentration**	(b)	**0.25X**	**1X**	**2X**
	**1mg/ml**	4.0±0.9	14.1±4.0	24.8±4.5
	**2mg/ml**	13.0±0.9	35.8±8.7	42.0±7.1
	**4mg/ml**	72.1±0.6	89.2±9.1	97.9±11.9
	**8mg/ml**	171.1±20.3	193.9±17.7	247.3±15.5

(a)Concentration of thrombin in NIH units of activity per ml for all fibrin hydrogel conditions (b) G’ values in Pa for various fibrinogen concentrations and all three cross-linking ratios, at a frequency of 0.5 Hz.

### Bi-clustering for Network Identification

**Figure 2:**
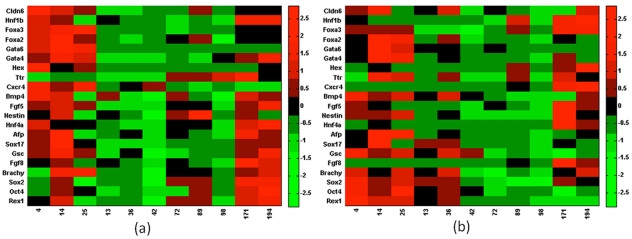
Effect of substrate stiffness on differentiation of embryonic stem cell to early germ layers. Embryonic stem cells were differentiated on fibrin substrates of varying stiffness and subsequently analyzed for early germ layer markers. In the above figure x-axis represents the storage modulus of the synthesized fibrin gel (Pa); the y-axis represents germ layer specific markers. Endoderm germ layer was analyzed in more detail since the initial observation revealed the endoderm to be most responsive to changes in the substrate properties. Experiments were conducted under 2 different culture configurations: 2-dimentional (Figure 2a) and 3-dimentional (Figure 2b). The data was normalized by mean centering and variance scaling.

**Figure 3:**
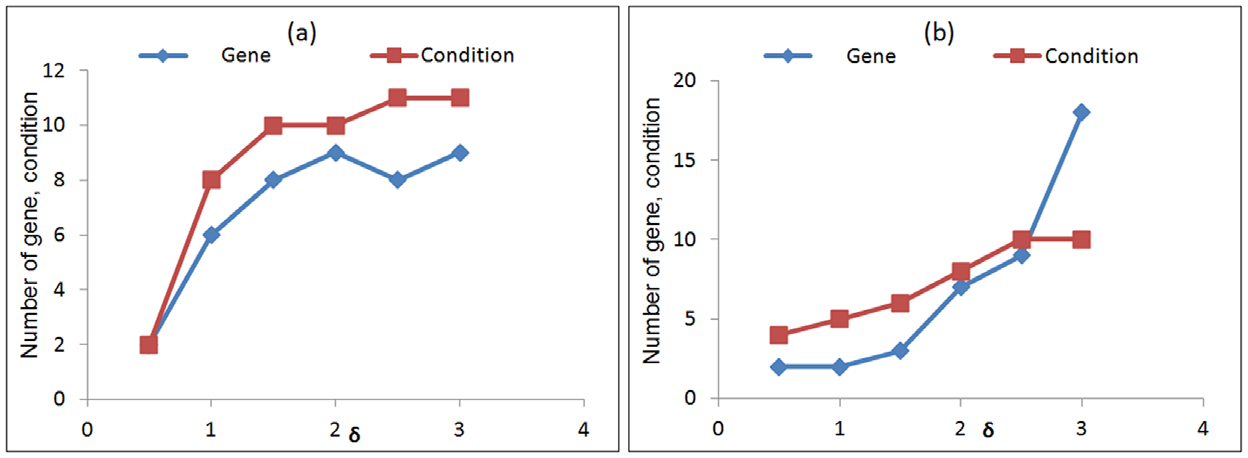
The effect of residue threshold, δ, on the number of genes and conditions in the optimal bi-cluster. The volume of the bi-cluster is highly sensitive to the prescribed residue on threshold. Increasing the threshold was found to increase the bi-cluster volume for both 2-dimensional (2-D) culture (a) and 3-dimensional (3-D) culture (b).

Our objective here is to analyze the regulatory interactions involved during mechanical induction of endoderm differentiation. The differentiated samples under the 12 different substrate conditions are analyzed for early germ layer markers, along with a more rigorous analysis of the endoderm markers. Figure (2) represents the differential gene expression levels for different substrate stiffnesses utilizing 2-dimensional (Fig. 2a) and 3-dimensional (Fig. 2b) cultures, respectively. If a specific network, consisting of certain transcription factors, becomes active under specific stimulation, it is expected that the participating transcription factors will show a coherent expression trend under those conditions. Hence identification of transcription factors exhibiting similar trend in expression across specific subsets of condition will elucidate the active network interaction. In this paper we have used the technique of bi-clustering to identify such information from the experimental gene-condition dataset. The bi-clustering formulation follows the structure proposed by Cheng and Church, where all possible gene-condition combinations are explored to minimize the residue. The residue is formulated to be a representative measure of the similarity of gene expression trends between different conditions, higher coherence of expression resulting in lower value of residue.

### Effect of Model Parameters on Bi-cluster

#### GA parameters

The bi-clustering algorithm formulated as an optimization problem is solved using Genetic Algorithm. The efficiency of Genetic Algorithm (GA) depends on the appropriate choice of the starting population along with other associated parameters. The initial population size plays an important role in the quality and efficiency of the algorithm and accordingly, a small population size results in local convergence or requirement of large generations. To avoid this, a population size of 20 was chosen, and the algorithm evolved for 500 generations at which point the solution remained unaltered. A crossover probability of 0.5 and mutation probability of 0.02 was chosen to maintain diversity in the population.

#### Bi-cluster parameters

Formulation of the bi-cluster identification problem as an optimization problem introduces multiple user defined parameters into the system. It will be important to understand the sensitivity of these parameters and evaluate its effect on the quality of the bi-cluster.

**Figure 4:**
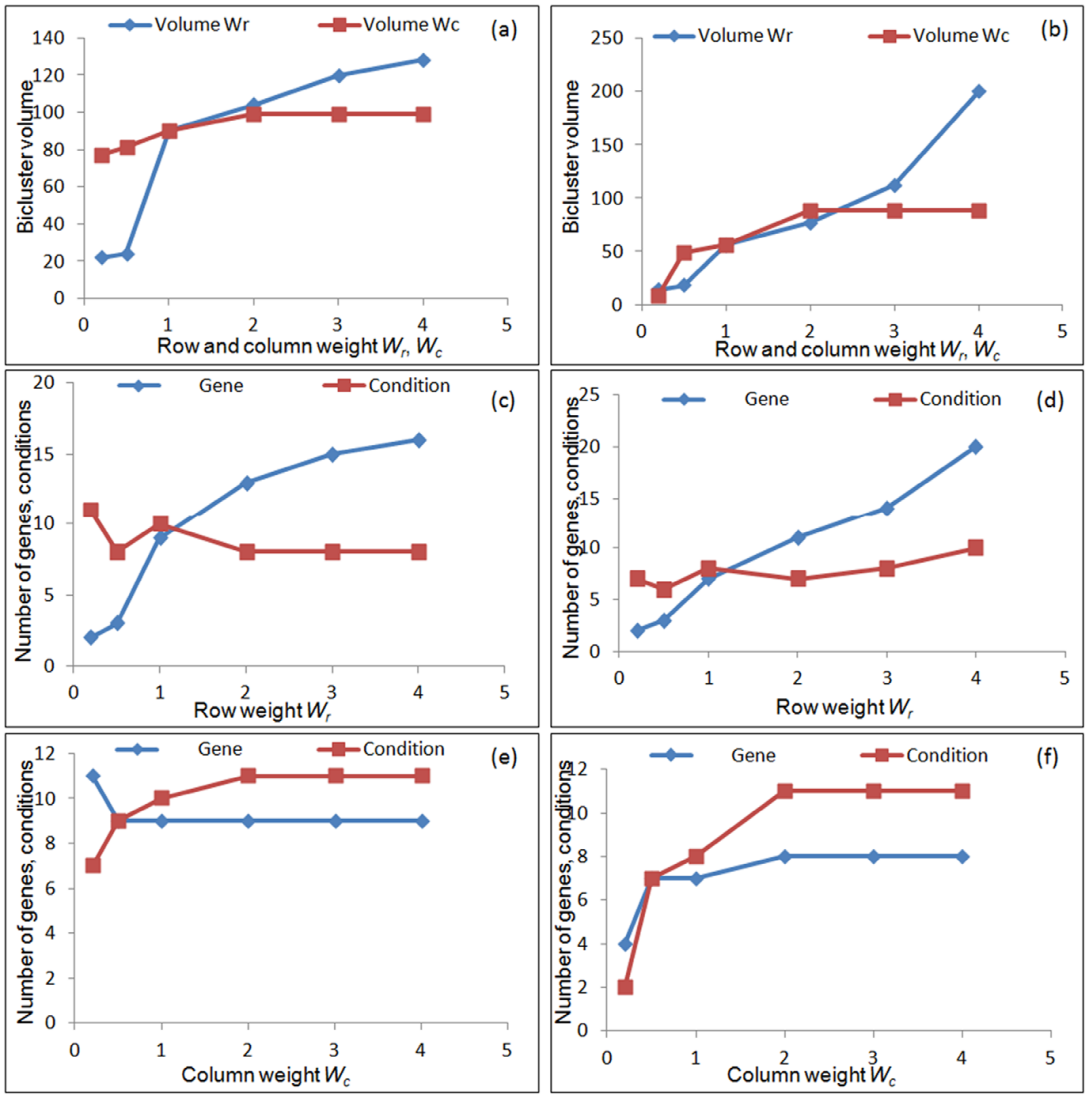
Effect of model parameters on features of optimal bi-cluster. (a-b) Variation of bi-cluster volume with change in row and column weights (*W_r_, W_c_*) for 2-D (a) and 3-D (b) experiments. In both cases changing *W_r_* was found to change the bi-cluster volume considerably, while it was less sensitive to changes in *W_c_*. The bi-cluster volume was further analyzed separately as rows and columns depicting genes and conditions. (c-f) Variation of number of genes and conditions in the optimum bi-cluster as a function of row weight (c,d) and column weight (e,f) for 2-dimensional culture (c,e) and 3-dimensional culture (d,f). This indicates the possibility of tailoring the bi-clusters by biasing the analysis towards genes or conditions by modifying the row and column weights.

**Figure 5:**
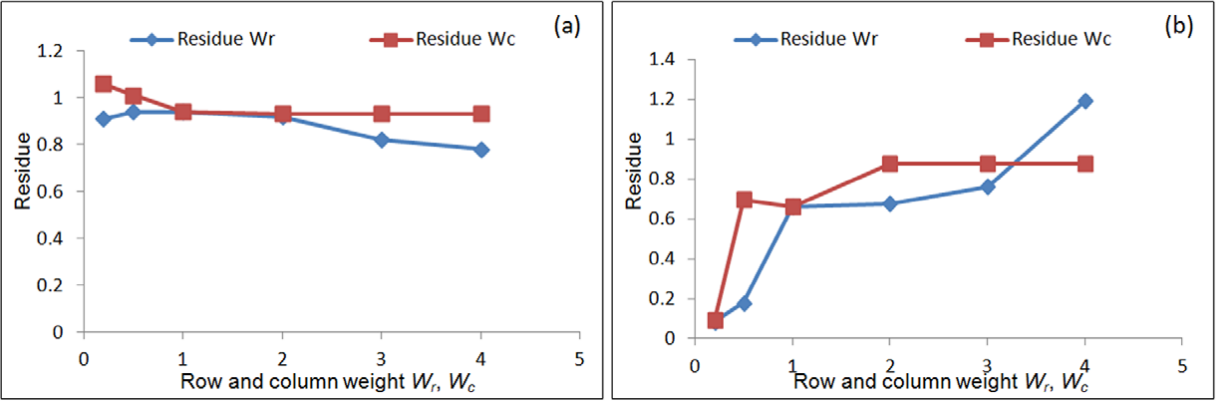
Variation of residue as a function of row and column weight for 2-dimensional culture (a) and 3-dimensional culture (b). For the 2D culture the residue was relatively insensitive to the row and column weights. For 3D culture it was possible to reduce the residue significantly by lowering row and column weights, but the resulting bi-cluster was also of a small volume and not useful for subsequent analysis. The residue however never exceeded the designated threshold, even for higher values of *W_r_* and *W_c_*.

The formulation of fitness function involves some free variables: δ - the threshold for residue; *W_v_, W_r_ and W_c_* – individual weights associated with the volume, row and column of the bi-cluster, respectively. These user defined parameters significantly affect the derivation of the optimal bi-cluster, the effect of which is evaluated for both the experimental data sets corresponding to 2-D and 3-D culture configurations (Figures 3, 4, 5). These weights allow the user to bias the bicluster to include more genes or conditions, based on the nature of the system under consideration. If it is expected that a relatively small set of transcription factors are co-regulated, but for a large number of stimulatory conditions one can bias the bilcuster to include more conditions than genes and vice versa.

Overall it is observed that both the culture configurations elicit approximately similar response with respect to the parameter values. Figure (3) represents the effect of threshold value δ on the number of genes and conditions constituting the bi-cluster. Increasing the value of δ increases the volume of bi-cluster. For a small value of δ = 0.5, the algorithm only identifies 2 genes and 2 conditions, while increasing δ to 1 gives a more reasonable bi-cluster of 6 gene and 8 condition for the 2-dimensional (2-D) case. Further increase of δ to 1.5 increases the bi-cluster to 8 genes and 10 conditions, which does not change appreciably with further increase in δ. For the 3D data set the response is more subtle for the lower ranges of δ, which had to be increased to 2 for identification of a larger volume of the bi-cluster.

The other parameters in the fitness function are the weights associated with the bi-cluster volume, rows and columns. Figure (4a–b) illustrates how the volume of the identified bi-cluster varies with the relative weights *W_r_* and *W_c_*. For both the 2-D and 3-D dataset it is observed that the volume of the bi-cluster is overall more sensitive to the row weight *W_r_* as compared to the column weight *W_c_*. While changing *W_r_* from 0.5 to 3.0 increases the bi-cluster volume from 20 to 120, an equivalent change in *W_c_* only changes the volume from 80 to 100. It is worth observing that lowering the value of *W_c_* does not appreciably reduce the volume of the bi-cluster. Similar effect was also observed in the 3D data set with the bi-cluster volume being more sensitive to *W_r_* than *W_c_*. To further analyze the effect of the weights, the bi-cluster volume is split up into the number of genes and conditions in Figure (4c–d) and (Fig. 4e–f) to compare the effect of *W_r_* and *W_c_*, respectively. Consistent with Figure (4a), the effect of *W_r_* is seen to be more prominent than *W_c_* for both genes and conditions. Figure 4c–d further shows that number of genes is more sensitive to *W_r_* than number of conditions. Increasing row weight increases the number of genes while reducing the number of conditions however, since the increase in number of genes is more dominant the overall effect is an increase in volume with *W_r_*. An opposite trend is observed for *W_c_*, where increase in *W_c_* increases the number of conditions and reduces the number of genes. However here the reduction in the number of genes is more subtle, hence the dominant effect is still an increase in volume, but much lower in magnitude than *W_r_*. For the 3-D case though both genes and conditions increased with increasing *W_r_* and *W_c_*. This analysis indicates the flexibility of including more genes in the bi-cluster by relaxing some of the model parameters, but the system is more rigid with respect to experimental conditions. It can be interpreted from here that of the 12 different substrate conditions there is only a restricted range of conditions in which a specific transcriptional network is getting activated.

In all the above analysis the threshold value (δ) for the bi-cluster was kept fixed at 1.5. However there is no rigid constraint in the formulation which prevents the residue from increasing. Since increase in the residue compromises the quality of bi-cluster, it is important to verify the range of residue attained by changing row and column weights. Figure (5) illustrates the effect of row and column weights on the residue of the bi-cluster for both 2- and 3-dimensional configurations for a fixed threshold value of δ at 1.5. In 2-D culture it was observed that changing either the row or column weights did not alter the residue appreciably, even though Figure (4) illustrates a significant increase in bi-cluster volume in response to increased *W_r_* and *W_c_*. The 3-D configuration was found to be more sensitive to *W_r_* and *W_c_*, where reducing the weights could significantly lower the residue of the identified bi-cluster. This comparison clearly indicates that the actual sensitivity is largely dependent on the experimental data set. It is also worth mentioning that the residue never exceeds the threshold δ even in the absence of an explicit constraint implementing the threshold. Overall this indicates that the quality of the bi-cluster is always preserved in our operating range of parameters. Quite encouragingly, the residue was relatively insensitive to changes in model parameters in the optimal range, which increases confidence on the identified interaction as having biological significance and not a numerical artifact.

Following the analysis above, we chose the value of δ = 2 in order to capture a reasonable volume of the bi-cluster. The weights *W_v_*, *W_r_* and *W_c_* are all chosen to be on the lower end of 1 in order to not bias the algorithm in the absence of any *apriori* information.

### Identification of Robust Bi-cluster

**Figure 6:**
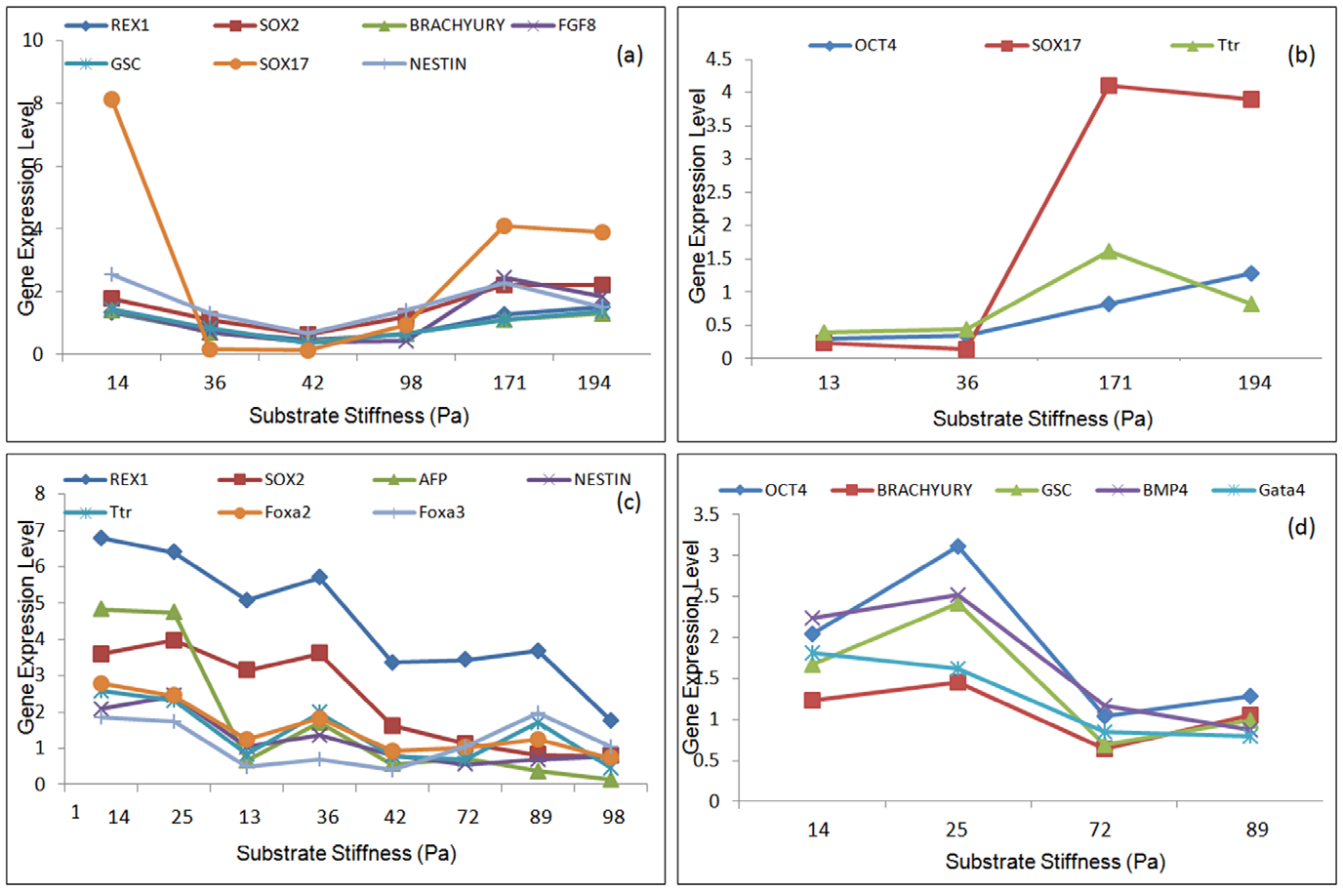
Optimal bi-clusters identified at mean value of experimental dataset. Solving equation (5) at mean value of the experimental data identifies the optimal bi-clusters for 2-dimensional culture (a, b) and 3 dimensional culture (c, d). The bi-clustering algorithm was solved sequentially by penalizing the previously identified bi-cluster in subsequent runs to avoid significant overlap. Single overlap was identified in the 2D dataset (Sox17) while no overlap was identified in the 3D dataset.

The bi-clustering problem is solved first at the mean value of the experimental data points. The present formulation for bi-clustering allows for overlaps through the penalty function, by sequentially penalizing the identified bi-clusters in repeated simulations. The concept behind overlapping comes from the understanding that the same transcription factor can be participating in multiple regulatory pathways. While a single bi-cluster indicates the co-regulation of sets of genes in one network, partial overlapping of two bi-cluster allows identification of transcription factors participating in multiple network pathways. Figure 6 illustrates 2 representative bi-clusters for 2-dimensional (Fig. 6a,b) and 3-dimensional (Fig. 6c,d) configurations, depicting the trend of co-regulated gene expression dynamics across the identified substrate conditions also outlined in Table 1. For the 2-D data set Sox17 is showing up in subsequent bi-clusters indicating Sox17 to be participating in different pathways. No such overlap, however, was observed between the 3-D bi-clusters. Analysis of the experimental data at its mean value identified significant co-regulation among different transcription factors, spanning across the three germ layers along with pluripotency markers. For example, the first bicluster of the 2D data set identified Sox17 (endoderm), Gsc (mesoderm), Nestin (ectoderm) in the same bi-cluster. The second bi-cluster identified Oct4 (pluripotency) and Sox17 in the same bi-cluster. Similar trend was also observed in the 3D data set, where the first bi-cluster includes mostly endoderm markers along with ectoderm marker Nestin. The second bi-cluster includes many of the mesoderm and mesendoderm markers along with pluripotency marker Oct4.

As mentioned earlier, the above bi-clusters are obtained at the mean value of gene expression data. Biological systems, more so embryonic stem cell systems, are subject to significant variability arising from system heterogeneity and stochasticity along with experimental errors. Before exploring the biological relevance of the bi-clusters represented in Figure 6, it will be important to evaluate the robustness of the algorithm and the identified bi-clusters based on the variability of the experimental dataset. Actual experimental repeats alone being insufficient in statistical analysis of such variability, the bootstrapping technique is adopted for the analysis and identification of a robust bi-cluster.

By bootstrap re-sampling a larger artificial data set is generated based on the existing limited experimental data of gene expression levels corresponding to specific substrate conditions. Bootstrapping is an efficient technique of determining robust solutions from limited experimental data-points, which typically is the case in biological systems. While it still extracts the information from the actual experimental replicates, it allows an estimation of subsequent experimental repeats without actually performing the experiments. Having obtained the bootstrap samples, the bi-clustering algorithm is applied at each of the bootstrap data points, to determine the optimum bi-clusters for each of the bootstrap samples. This procedure results in an entire array of gene-condition bi-cluster which will then be analyzed for the identification of robust bi-cluster.

**Figure 7:**
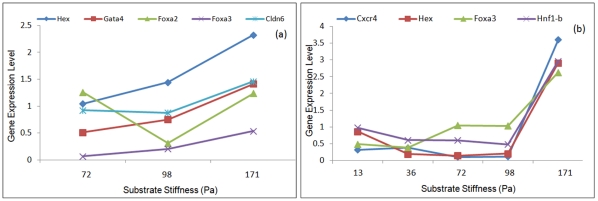
Identified robust bi-clusters. Robust bi-cluster for 2-dimensional culture (a) and 3-dimensional culture (b). Bootstrap re-sampling of the experimental data set, followed by biclustering at the bootstrap point leads to the identification of the robust bi-cluster. These bi-clusters are insensitive to experimental noise and appear with high frequency in the bootstrap analysis. Note that the robust bi-cluster is different from that identified at the mean.

It was expected that a robust gene-condition bi-cluster will be repeated significant number of times over the array of bi-clusters generated from the bootstrap data. Surprisingly, analysis of the bi-cluster array did not reveal any such highly repeated bi-cluster, the highest repeat being less than 10% over the entire random trials. Instead of the entire bi-cluster, what was found to be conserved over a large population of the array were subsets of the gene-condition bi-clusters. Thus instead of an entire bi-cluster being repeated multiple times, only a portion of it was found to be appearing in subsequent repeats. This indicates that each bi-cluster has some noise in it which needs to be excluded in subsequent analysis. It is reasonable to suggest that the portions of the bi-cluster with high number of repeats constitute a robust bi-cluster. Figure (7a) illustrates the 5-gene 3-condition bi-cluster appearing almost 70% times in the analysis of the 2-dimensional data array. A similar analysis in the 3-dimesional data set identifies a 4-gene 5-condition bi-cluster appearing the highest number of times, which is illustrated in Figure (7b). It is important to note that neither of these bi-clusters alone was identified in any of the data set, instead they always appeared as a subset of the identified bi-cluster which constituted additional genes and conditions. Since the rest of the bi-cluster was not being repeated in the bootstrap analysis it is reasonable to conclude that those are spurious connections resulting from noise in the experimental data. The bi-clusters illustrated in Figure 6 however was not affected by the noise and kept appearing in most of the bootstrap repeats.

### Effect of Model Parameters on Robust Bi-cluster

**Figure 8:**
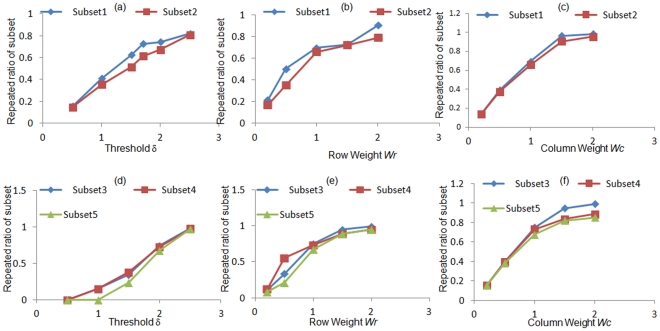
Sensitivity of the identified robust bi-cluster on model parameters. Bi-cluster of the bootstrap data identifies 2 robust bi-clusters for 2-dimensional dataset and 3 robust bi-clusters of the 3-dimensional dataset. Figures (a-c) illustrates the frequency of repeats of the robust bi-clusters to threshold d (a), row weight *W_r_*(b) and column weight *W_c_*(c) for 2-dimensional culture; Figures (d-f) represents the same for 3-dimensional culture.

As discussed earlier the quality of optimum bi-cluster depends considerably on the chosen parameters involved in the formulation of the optimization problem. In order to determine the sensitivity of the model parameters on the robust bi-cluster obtained after bootstrapping, the entire bootstrap and bi-cluster simulation is repeated at different parameter values and the frequency of occurrence of the identified robust solution is determined. Instead of considering only the most repeated bi-cluster, multiple subsets were considered to assess the generality of the analysis. Figure (8) illustrates the frequency of repeat of these solutions for different values of threshold δ assigned to the residue, for both 2- and 3-dimensional culture configurations. The details of the solution are as follows: 2-Dimensional culture: Subset1 – Hex, Cldn6, Foxa2, Foxa3; Subset 2 – Hex, Cldn6, Foxa2, Foxa3, Gata4 both for stiffness values of 72 Pa, 97.9 Pa and 171 Pa; 3-Dimensional culture: Subset 3 – Cxcr4, Hnf1b, Foxa2; Subset 4 – Cxcr4, Hnf1b, Hex; Subset 5 - Cxcr4, Hnf1b, Foxa3, Hex all three for stiffness values of 13 Pa, 42 Pa, 72 Pa, 97.8 Pa and 171 Pa. These above bi-clusters indicate that sets of transcription factors which are being consistently co-regulated over specific substrate stiffness conditions. For both culture conditions it is confirmed that the robust bi-cluster appears more than 50% of time for δ values of 1.5 and higher. For δ value of 1 and less the bi-cluster is repeated less than 40% of time, since at such low values of the threshold the average size of the bi-cluster is typically lower than that of the robust bi-cluster. Similar trend was observed while varying *W_r_* and *W_c_*, for values of 1 and above the robust bi-cluster appeared more that 50% of time. Values of 0.5 and less showed less than 40% appearance of the robust bi-cluster, resulting from the small size of the bi-cluster at lower values of *W*_r_ and *W_c_*. Hence it can be concluded that the identified robust bi-cluster is robust against experimental noise as well as model parameters. A closer look at the identified robust bi-clusters revealed that fibrin substrates in the mid-range stiffness values are typically acting in synergy. This is true both for the 2-D and 3-D culture configuration, although for 3-D, the range extends to lower stiffness values as well. Quite interestingly bi-clusters including both low and high stiffness values were largely absent perhaps indicating different transcriptional networks are dominating in different substrate stiffness regimes.

## Discussion

The effect of mechanical microenvironment on stem cell fate commitment is being increasingly appreciated and researched intensely following the report by Disher *et al*. [3]. While some mechanistic study to understand the mediators of such response has been initiated [22], the transcriptional response as a result of such mechanical induction has not been analyzed yet. In our previous work we reported the effect of fibrin substrate mechanical properties on early differentiation of mouse embryonic stem cells [1]. It was observed that substrates of lower stiffness values were preferentially favoring endoderm differentiation. In this report we investigate the network interaction of relevant endodermal genes in the process of mechanically induced differentiation.

In our experimental system mouse embryonic stem cells were differentiated for 4 days on fibrin substrates fabricated with 12 different conditions. At the end of the experiment the differentiated cells were analyzed in details for expression levels of endoderm related markers for all the 12 substrate conditions. Towards identification of prospective networks of interactions from this gene-condition data set, we are using the bi-clustering algorithm to identify sets of genes having similar patterns of response over specific substrate conditions, and hence can be considered to be co-regulated. Following the report by Divina [16] the bi-clustering algorithm is formulated as an optimization problem, and solved using evolutionary strategy. The problem of finding the minimum set of bi-cluster, either mutually exclusive or overlapped, has been shown to be NP-hard [11]. Such class of problems is particularly well suited for evolutionary algorithms because of the inherent exploratory nature of the algorithm, which enables searching the entire space and escaping local minima. Use of evolutionary algorithm suffers from the criticism of lack of convergence criterion; however this is not expected to be critical for the present application. A sub-optimal bi-cluster which adequately satisfies the threshold requirement should still identify sets of co-regulated genes. However we are ensuring to evolve the algorithm for sufficiently high generations to identify a near-optimal solution. The GA parameters are also chosen carefully to ensure diversity of population and avoid local convergence.

While bi-clustering allows identification of sets of genes co-regulated under specific sets of conditions, it is difficult to comment on its robustness in the presence of data or system variability. In order to increase our confidence on the identified bi-cluster, we adopted the bootstrap re-sampling technique to generate a larger data set from the limited experimental repeats. The bi-clustering algorithm was subsequently solved at each of the bootstrap sample points and the data analyzed for identification of a robust bi-cluster. While the robust bi-cluster was determined by bootstrapping in the face of experimental noise, it will be interesting to investigate its robustness to the chosen model parameters as well. Hence we repeated the entire process of bi-clustering and bootstrapping at different values of parameters and tested the frequency of occurrence of the robust bi-cluster in those repeats. As illustrated in Figure 8, the identified robust bi-cluster was highly repeated under different ranges of model parameters.

**Figure 9:**
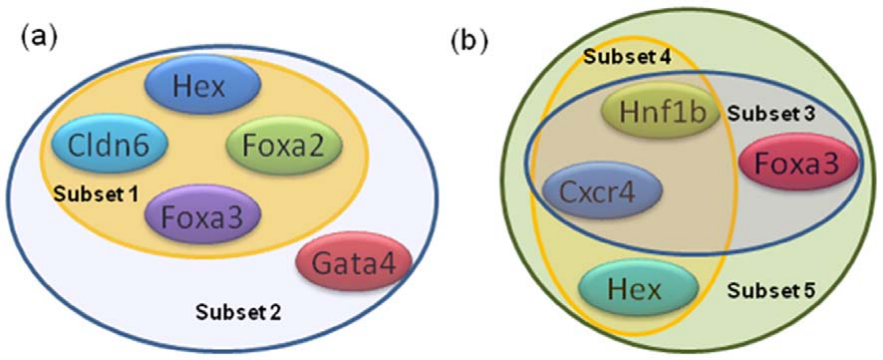
Robust subsets of co-regulated genes. Substets of co-regulated genes for 2-dimensional (a) and 3-dimensional (b) culture configuration. The 2D data set identifies two subsets while 3D data set identifies 3 subsets of regulatory interactions.

Figure 9 represents the subsets of transcription factors identified to be robustly co-regulated during mechanical induction of stem cell differentiation in 2-dimensional (Figure 9a) and 3-dimensional (Figure 9b) culture configuration. The fibrin gel conditions identified in both 2-D and 3-D configurations was in the mid-range of stiffness; 72 Pa–193.9 Pa for 2-D and 13 Pa–171 Pa for 3-D. Absence of co-occurrence of substrate conditions in the extreme ranges perhaps indicate a significantly different transcriptional network in action based on substrate stiffness range. It is important to note that the current analysis primarily concentrates on endoderm related transcriptional network which may be more prominent in the mid-range of the substrate stiffness considered.

In order to understand how the identified network interaction compares with existing knowledge of endoderm regulation we performed a comprehensive review of literature. Quite interestingly many of the current identified interaction for mechanically induced endoderm have also been observed either in endoderm or endoderm derived organs. Cldn6, Foxa2, and Gata4 are markers used to identify definitive endoderm and gut tube development [23], [24]. Foxa2 (HNF3β) and Foxa3 (HNF3γ) along with Foxa1 (HNF3α) were first identified as regulators of liver genes [25], [26], [27]. Other than liver, Foxa2 and Foxa3 have been found to be co-expressed in a number of endoderm derived tissues including midgut, stomach, pancreas, adrenal tissue and hindgut at different stages of development. Moreover Foxa2 and Foxa3 are required for efficient expression of the gene that encodes for pancreatic α amylase [28]. Hex expression is also present in the definitive endoderm and is necessary for proper liver development [29]. It also co-expressed with Foxa2 during liver development and it has been shown that Hex is transactivated by Gata4 and Foxa2 [30]. Other than their interactions in liver, Foxa2 and Gata4 have been found to be expressed in other tissues, in particular, in the jejunum [31]. Gata4 has been established to be directly regulated by Foxa2 and therefore implicated in the establishment of a Gata4 expressing population that directs the development of the definitive endoderm [32]. Other non-endodermal interactions have been also found between some of these genes. In particular, Gata4 and Hex both participate in cardiogenesis [33].

For the network interactions identified for 3-dimentional culture, Hex, Foxa3 and Hnf1β are all liver markers and involved in liver differentiation at several stages of development. Foxa3, along with Hnf1β are spatio-temporally co-expressed in the liver during development. In adult liver, Hnf1β and Foxa3 are found in the hepatocytes, while not present in the bile ducts. During oval cell differentiation, however, both factors are co-expressed at similar levels in hepatocytes, oval cells, intestinal glands and foci [34]. Also, expression of these 2 factors is higher in hepatocytes that are in close proximity to portal veins in the liver [34]. With respect to stem cell differentiation, isolation of Hex and Cxcr4 expressing cells from differentiating embryonic stem cells results in a population expressing anterior definitive endoderm markers. These cells have been expanded and differentiated toward liver and pancreatic fates [35].

Correlating such information with the co-regulation information extracted using biclustering methodology indicates somewhat different patterning of differentiation between the 2-dimensional and 3-dimensional culture conditions. While the regulatory information obtained from 3-dimensional culture is more indicative of endoderm to hepatic differentiation, analysis of the 2-dimensional culture indicates a more heterogeneous potential to different endoderm derived tissues. Hence 3-dimensional culture in fibrin gels may be better suited for hepatic maturation. For other endoderm-derived tissue the 2-dimensional culture may have stronger potential, but this may require augmentation by growth factors for specificity in differentiation.

## Materials and Methods

### Fibrin Gel Synthesis

Fibrin hydrogels comprising 1, 2, 4, and 8 mg/ml of fibrinogen were synthesized. The fibrinogen to thrombin ratios of 10, 2.5, and 1.25 mg/U (fibrinogen/thrombin) were synthesized for each fibrinogen concentration as previously described [35]. For convenience these ratios are referred to as 0.25X, 1X, and 2X respectively throughout the text. Total of 12 different substrate conditions were used in the current study.

### Mechanical Characterization of Fibrin Gels

Gel discs of 35 mm diameter, prepared as described for 2D gel synthesis, were deposited onto glass slides which were pre-rinsed with DI water. The samples were then allowed to gel fully at 4°C. After complete gelation, they were fully immersed in the same media used for differentiation studies. The glass slides were then secured to the Peltier cell of a TA Instruments AR2000 stress-controlled rheometer, which was kept at 37°C throughout the measurements.

A frequency sweep was then performed, using a 25 mm stainless steel in parallel plate geometry with sandpaper glued to the plate to avoid slippage. The samples were subjected to an oscillatory train described by equation (1), where γ_0_ is the amplitude of the oscillatory strain (5%), f is the frequency and t is the time. Frequencies employed ranged from 0.1 to 100 rad/s.
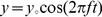
(1)The stress required to achieve the specified strain was measured and the components of the complex modulus, the storage (G’), and loss (G”) moduli were accordingly determined.

### Propagation of Embryonic Stem Cells

Murine ESD3 cells (ATCC) were cultured in knock-out Dulbecco’s modified Eagle’s medium (DMEM; Life Technologies Inc.) supplemented with 15% replacement serum, 4 mM L-glutamine (Cambrex, Walkersville, MD, USA), 100 U/ml penicillin (Life Technologies), 100 U/ml gentamicin (Life Technologies), 1000 U/ml leukemia inhibitory factor (LIF; Chemicon International, Temecula, CA, USA) and 0.1 mM2-mercaptoethanol (Life Technologies) on gelatin-coated T75 tissue culture flasks. Cells were cultured at 37°C and in a 95% air/5% CO2 atmosphere.

### Differentiation of Embryonic Stem Cells

The ESCs were induced to differentiate by culturing them in fibrin substrates of varying mechanical properties, modified by altering the fibrinogen concentration and cross-linking ratio. The mESCs were differentiated in two culture configuration, 2-dimensional – where the cells are seeded on top of preformed gels and 3-dimensional – where the cells are embedded inside the gel. For both cases, the cells were maintained in DMEM medium (Invitrogen) supplemented with 10% FBS, 4 mM L-glutamine (Cambrex) and 100 U/ml penicillin, with media being changed every day. The differentiated cells were analyzed for their germ layer commitment by qRT-PCR for relevant markers.

#### Cell culture in 2D

For differentiation of the ESCs on fibrin substrate, the cells were tripsynized, washed and replated in appropriate configurations. For the 2D culture 30,000 cells in 200 ul media were plated on top of the pre-formed fibrin gels prepared on wells of 48 well plates and polymerized overnight at 4°C temperature.

#### Cell culture in 3D

For 3D cell culture format 100,000 cells were re-suspended in the fibrinogen solution before adding thrombin and plated on wells of 48 well plates. The gel with the entrapped cells was then allowed to polymerize for one hour at 4°C temperature, after which the culture media was added and subsequently the culture was incubated.

### qRT-PCR Analysis

ESCs cultured in the two- or three-dimensional configuration were harvested by trypsin after five days of differentiation and RNA was extracted using NucleoSpin kit according to the manufacturer’s protocol. The sample absorbance at 280 nm and 260 nm was measured using a BioRad Smart Spec spectrophotometer to obtain RNA concentration and quality. Reverse transcription was performed using ImProm II Promega reverse transcription kit following the manufacturer’s recommendation. qRT-PCR analysis was performed for pluripotency and early germ layer markers.

The cycle number at the threshold level of log-based fluorescence is defined as Ct number, which is the observed value in most real-time PCR experiments, and therefore the primary statistical metric of interest. ΔCt is equal to the difference in threshold cycle for target and reference or control (ΔCt = Ct_target_−Ct_reference_). ΔΔCt is equal to the difference between ΔCt_sample_ and ΔCt_control_ (ΔΔCt = ΔCt_sample_−ΔCt_control_). The fold change of a target gene is defined by.
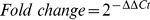
(2)Total of 12 different substrate conditions were used for differentiation. The ESCs differentiated at each of these 12 conditions were analyzed for 21 markers: Rex1, Oct4, Sox2 (pluripotency); Brachyury T, FGF8, GSC (mesoderm); Nestin, FGF5, BMP4 (ectoderm); Sox17, AFP, HNF4, Cxcr4, Ttr, Hex, Gata4, Gata6, Foxa2, Foxa3, Hnf1-b, Cldn6 (endoderm). qRT-PCR analysis was repeated in triplicate.

### Bi-clustering Formulation

In this report the bi-clustering problem is formulated as an optimization problem, following the report by Divina [16]. The objective of bi-clustering is to identify subsets of genes which exhibit similar patterns of expression trend across specific conditions. It is important, however, to eliminate the redundant case of negligible change in expression levels across different conditions. The objective thus is to determine largest subsets of matrices with (i) low mean squared residue (ii) high row variance (iii) low levels of overlapping among bi-clusters. The details of the formulation is discussed in [16] and briefly summarized below.

Mean squared residue of the bi-cluster (I,J) is defined as.
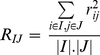
(3)Where *r_ij_* is the residue of an entry *e_ij_*of the bi-cluster (I,J) defined by
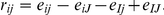


*e_iJ_* is the base of gene *g_i_* given by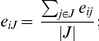 *e_Ij_*is the base of condition *c_j_* given by 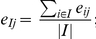 the base of the bi-cluster is mean of all entries of bi-cluster (I,J) given by 
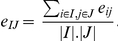


The residue can be viewed as the degree of coherence between elements in the bi-cluster, lower residue indicating stronger coherence. The quality of the bi-cluster is thus assessed by the mean squared residue, lower value of which indicates better quality of the bi-cluster. The optimization problem is formulated to obtain a bi-cluster with the mean squared residue value lower than a predefined threshold δ. The trivial bi-clusters are eliminated by considering the row variance, defined by.
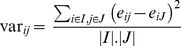
(4)The overall objective thus is to determine bi-clusters of maximum size, with the residue lower than predefined δ, exhibiting high row variance and low overlap between different bi-clusters. The fitness function is thus formulated as [16]:
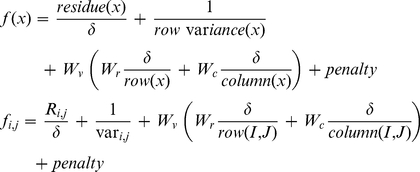
(5)In the above formulation the first term on the right represents the mean squared residue which is desired to be lower than user specified threshold δ, which leads to the first term being less than 1. The second term representing the row variance ensures that the bi-cluster is including genes with interesting dynamics, instead of trivial solutions. The third term of the fitness function represents the volume of the bi-cluster and allows some flexibility to bias the optimization routine towards favoring genes or conditions in the bi-cluster. 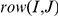 and 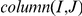 represents the number of rows and columns respectively in the bi-cluster 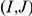.Wv,Wr and Wc are relative weights assigned to the volume, rows and columns of the bi-cluster respectively, as a measure of their relative importance. The penalty term in the fitness function is designed to reduce overlap between bi-clusters. The penalty is evaluated as
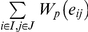
where the weight 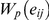
for each element 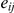
of the expression matrix is:
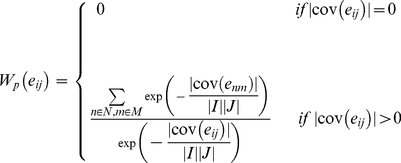
(6)where *N* and *M* are the number of rows and columns present in the expression matrix, and 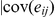 represents the number of identified bi-clusters containing the element eij. Use of the penalty term biases the search against elements which already appeared in previous bi-clusters, hence reducing overlapping.

### Solution Procedure

The bi-clustering problem has been identified to be NP-hard, which can efficiently be handled by evolutionary algorithm. Following the report by Divina [16], we address the bi-clustering problem by genetic algorithm (GA), which has been proven to have an excellent performance on highly complex optimization problems [36], [37], [38].

Genetic Algorithm (GA) [39] is a population-based stochastic iterative optimization technique based on Darwinian concepts of evolution. It represents a class of search and optimization procedure that are patterned after the biological process of natural selection. In GA each optimization variable is typically encoded as string of binary bits, which are appended together to form a chromosome. In present formulation each chromosome consists of *N* binary bits for genes and *M* binary bits for conditions, resulting in *N+M* chromosome size. Assigned value of 0 or 1 in the binary string will dictate absence or presence of the corresponding gene or condition in the bi-cluster. Hence each individual of the GA population directly represents a candidate bi-cluster. For example, for a case of 7 genes and 5 conditions, an individual represented by: 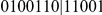 consists of genes 2, 5, 6 and conditions 1, 2 and 5 as a prospective bi-cluster.

Each chromosome can be directly decoded to evaluate the parameter values and objective function, also called the fitness function. The solution procedure is initiated by randomly assigning a population of chromosomes. This population is continuously evolved by GA operators: reproduction, crossover and mutation, to create new and better populations. This procedure is repeated until a predefined termination criterion is satisfied. For the present study the simulation was allowed to run sufficient generations until no significant change in the objective function was observed. It is worth noting here that such a procedure lacks guarantee of optimality, which is a common criticism for genetic algorithm.

### Determination of Robust Solution

While biological samples are inherently of uncertain nature, stem cell systems are notorious for their heterogeneity, making analysis and interpretation of data particularly challenging. Hence a bootstrap technique has been adopted to determine a robust set of co-regulated genes constituting a network. The basic idea of bootstrapping is to generate a large data set by re-sampling a smaller sample of the original data set, under the assumption that the sample is a good representation of the system. Typically the re-sampling is done with replacement, indicating that the sampled data is returned back to the original data set, allowing it to be sampled again in subsequent draws. Bootstrap re-sampling technique is most commonly applied in the area of nonlinear regression, to determine a robust confidence interval of parameters in a data-lean scenario.

For example, for a dynamic system with parameter vector ***q***, if the true model response with respect to time ***t*** is denoted by 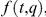 collecting experimental data at discrete time intervals will result in data points 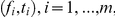 with 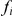 representing the collected data at each time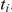 Each data point will be associated with a measurement error 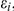 given by 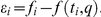 Given that the error is statistically independent with a common distribution [40], the bootstrap technique can be performed in two variants. The first one re-samples the original data set 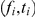 in generating the desired bootstrap points. The second variant re-samples the residue given by 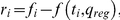 where ***q_reg_*** is the estimated parameter obtained by regression using the original dataset.

The present application follows a similar format of re-sampling using the first procedure of sampling the original dataset using Monte Carlo algorithm to determine the bootstrap sample. Instead of having samples at discrete time points, we draw our samples under distinct experimental conditions. Given experimental data set of 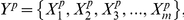 where the superscript *p* represents the experimental repeats, *m* represents total number of experimental conditions. Each element 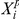 is a vector given by: 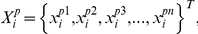 where *n* represents the number of genes analyzed at each conditions and for each experimental repeat. The bootstrap re-sampling is generated by randomly drawing from the *p* repeats for each of the *m* conditions, to generate 5000 sets of data points. When a particular 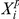 is sampled the entire array of gene expression is drawn from the same sample point.

In a typical regression problem after generating the bootstrap data set a regression is performed using the bootstrap data following which the estimated parameters are analyzed for its variance, confidence interval etc. The structure of the current problem however does not allow an analogous approach. In our approach an array of alternate bi-clusters is generated by solving the entire bi-clustering problem at each of the bootstrap data points. These bi-clusters are subsequently analyzed to identify a representative robust bi-cluster in the face of experimental uncertainty.
